# *Trypanosoma evansi* and Surra: A Review and Perspectives on Origin, History, Distribution, Taxonomy, Morphology, Hosts, and Pathogenic Effects

**DOI:** 10.1155/2013/194176

**Published:** 2013-08-19

**Authors:** Marc Desquesnes, Philippe Holzmuller, De-Hua Lai, Alan Dargantes, Zhao-Rong Lun, Sathaporn Jittaplapong

**Affiliations:** ^1^Cirad-Bios, UMR-InterTryp, Montpellier 34000, France; ^2^Faculty of Veterinary Medicine, Kasetsart University, Chatuchak, Bangkok 10900, Thailand; ^3^Center for Parasitic Organisms, School of Life Sciences, Sun Yat-Sen University, Guangzhou 510275, China; ^4^Central Mindanao University, Mindanao, Philippines

## Abstract

*Trypanosoma evansi*, the agent of “surra,” is a salivarian trypanosome, originating from Africa. It is thought to derive from *Trypanosoma brucei* by deletion of the maxicircle kinetoplastic DNA (genetic material required for cyclical development in tsetse flies). It is mostly mechanically transmitted by tabanids and stomoxes, initially to camels, in sub-Saharan area. The disease spread from North Africa towards the Middle East, Turkey, India, up to 53° North in Russia, across all South-East Asia, down to Indonesia and the Philippines, and it was also introduced by the conquistadores into Latin America. It can affect a very large range of domestic and wild hosts including camelids, equines, cattle, buffaloes, sheep, goats, pigs, dogs and other carnivores, deer, gazelles, and elephants. It found a new large range of wild and domestic hosts in Latin America, including reservoirs (capybaras) and biological vectors (vampire bats). Surra is a major disease in camels, equines, and dogs, in which it can often be fatal in the absence of treatment, and exhibits nonspecific clinical signs (anaemia, loss of weight, abortion, and death), which are variable from one host and one place to another; however, its immunosuppressive effects interfering with intercurrent diseases or vaccination campaigns might be its most significant and questionable aspect.

## 1. Introduction

Trypanosomes found in mammals (including humans) are blood and sometimes tissue parasites of the order Kinetoplastida, family of the Trypanosomatidae, genus *Trypanosoma*, principally transmitted by biting insects, in which most of them undergo a biological cycle. They are grouped into 2 sections: *Stercoraria*, which develops in the posterior part of the insect digestive tract, including *Trypanosoma cruzi*, both an extra- and intracellular parasite that is responsible for Chagas disease, a major human disease affecting 15 million people and threatening 100 million people in Latin America [1], and *Salivaria* which develops in the anterior part of the insect digestive tract, such as the main African livestock pathogenic trypanosomes, including the agents of sleeping sickness, a major human disease affecting around half a million people and threatening 60 million people in Africa [2]. African livestock trypanosomes are threatening 48 million cattle in an area of 10 million sq/km in 37 African countries [3]; they cause fever, anaemia, weakness, and nervous symptoms, responsible for major production losses (meat, milk, draught power, fertility, and manure), leading to cachexia and sometimes abortion and/or death in the absence of treatment. Animal Trypanosomoses are nowadays a permanent constraint for livestock in Africa, Asia, and Latin America, but their geographical distribution is still evolving.

The main African pathogenic trypanosomes belong to three subgenera of the salivarian section, namely, *Nannomonas* (*Trypanosoma congolense*), *Duttonella* (*Trypanosoma vivax*), and *Trypanozoon* (*Trypanosoma brucei* group). These parasites are mostly transmitted cyclically by the tsetse fly in which the procyclic forms undergo a cycle of transformations and multiplications leading to infective metacyclic forms, which may be inoculated by the tsetse flies with its saliva into a new host [4]. Due to this biological association, the geographical distribution of African trypanosomes is closely related to that of tsetse flies and then restricted to sub-Saharan Africa, approximately below 15° North. However, in some instances, in addition to the movements of their hosts, the geographical distribution of trypanosomiasis does not fit that of the tsetse fly, due to several other ways of transmission. Amongst them, while direct vertical, oral, sexual, and iatrogenic transmission may have an occasional impact, the most important alternative way is mechanical transmission by biting insects [5]. This way of transmission does not involve a specific biological relation between parasite and vector. In the absence of biological association and multiplication, pathogens are simply sampled from one host, transported to another, and inoculated with the saliva of the biting insect, prior to the absorption of blood [6]. By this means of transmission, some African pathogenic *Trypanosoma *species could spread not only outside the tsetse belt in Africa, but also towards other continents [4].

In the subgenus *Duttonella*, *T. vivax* is mechanically transmitted by tabanids and stomoxes, in both Africa [7, 8] and Latin America [9–11], with an increasing impact in cattle breeding. *Trypanosoma vivax* has not invaded Europe and Asia, so far, but its potential for geographical distribution is somewhat similar to that of *T. evansi* (in links with cosmopolitan mechanical vectors), though limited by a narrower host range compared to *T. evansi*. Indeed, *T. vivax* infects mainly bovines, and, to a lesser extent, horses [12, 13], so its potential for geographical spread and enzootic establishment would hardly compete with that of *T. evansi*.

In the subgenus *Nannomonas*, although *T. congolense* was suspected early [14, 15], or proved to be mechanically transmitted [16–19], the relatively low parasitaemia recorded in its main host (cattle) does not favour mechanical transmission, which results in rare epidemiological evidence for such transmission in the field [16, 20].

In the subgenus *Trypanozoon*, mechanical transmission of *T. brucei* spp. was described both through contamination by sucking flies and through serial biting action by biting insects such as tabanids and stomoxes [21, 22], including tsetse as mechanical vectors [23]. In the particular case of *Trypanosoma evansi*, due to a loss of genetic material, the parasite can no longer undergo its cycle in tsetse flies, thus it is mainly mechanically transmitted by biting insects, which probably selected parasites presenting the best ability for such transmission. For this reason, *T. evansi* spread outside the tsetse belt in Africa, towards the Middle East and Southern Asia, and was exported with livestock to Latin America, and even to Australia and Europe [4], although in the latter cases, early eradication was possible.

It is not only the transmission of *T. evansi* that is different from that of the other African trypanosomes, but also its capacity to invade a host's tissues (such as *T. equiperdum*). The most pathogenic African livestock trypanosomes, *T. congolense* and *T. vivax*, known as blood parasites, exhibit a direct relation between pathogenic effects and the presence of parasites in the blood. Although *T. evansi *can exhibit very high parasitaemia, especially in camels, horses, and dogs (and even occasionally cattle and buffaloes), it must be considered as both a blood and tissue parasite, due to its ability to invade the nervous system, not only in horses and and dogs but also in cattle, buffaloes, and pigs [24]. When the parasite is in very low numbers (although able to induce immunosuppressive effects), or when it is absent from the host blood stream (although present in the nervous system), identification of the etiological agent and evaluation of its pathogenic effects and impact are especially difficult. For these reasons, medical and economic impacts of *T. evansi* have most often been underestimated. Amongst other things, this review aims to provide a new view on this old parasite whose tendency to travel does not appear to be extinct!

## 2. Origin, History, and Geographical Distribution

*Trypanosoma *(*Trypanozoon*) *evansi* (Steel 1885) Balbiani, 1888, is the first pathogenic mammalian trypanosome to be described in the world, in 1880, by Griffith Evans, in the blood of Indian equines and dromedaries [4]. Its principal host is originally the camel but it is present in dromedaries, horses, and other Equidae as well as in a large range of other hosts.

*Trypanosoma evansi* is thought to be derived from *T. brucei brucei* (cyclically transmitted by tsetse flies), but it is no longer able to undergo its cycle in *Glossina* due to the loss of the maxicircles of kinetoplastic mitochondrial DNA [25–27]. When this phenomenon occurred is not known, and some authors even recently suggested that it might have occurred in several instances [27].

In Africa *T. evansi* is present in all countries where camels are present, north of a line extending from Senegal (15° North) to Kenya (equator), above the tsetse belt; it is found not only in Mauritania, Morocco, Algeria, Tunisia, Libya, Egypt, Sudan, Eritrea, and Ethiopia, but also in the northern parts of Mali, Burkina Faso, Niger, Nigeria, Chad, Somalia, and Kenya [4]. Nowadays, its geographical distribution is continuous from the northern part of Africa through the Middle East to South-East Asia.

Although it is not possible to date the initial spread of *T. evansi* eastwards, the analysis of historical data suggests that surra was already present in India since time immemorial, at least VIII centuries B.C., and that livestock must have suffered from it in the absence of treatment [4, 28]. It is present in sub-Saharan and Mediterranean climates but can be found in temperate areas as well as in arid deserts and semiarid steppes.

*T. evansi* is continuously present eastwards, in the Arabian peninsula, including Saudi Arabia, Oman, the United Arab Emirates, Jordan, Israel, Lebanon, Syria, Iraq, and Turkey, and even with one occasional record in Bulgaria; it is present from Iran to Kasakhstan as well as in Afghanistan and Pakistan [16, 20, 29–31]. Curasson (1943), quoted by Hoare [4], said that it is conceivable that surra was introduced beyond Africa by the ancient Egyptians since they used dromedaries in their military campaigns in Arabia, Mesopotamia, Persia, and Baluchistan.

*Trypanosoma evansi* is present in India, China, Mongolia, Russia (from Kuibyshev, 53°N, to the Caucasus, 44°N), Bhutan, Nepal, Myanmar, Laos, Vietnam, Cambodia, Thailand, Malaysia, the Philippines, and Indonesia [32, 33]. Its presence was suspected in Papua New Guinea but was not confirmed [34]. It is so far absent from Australia [33].

The extension of *T. evansi* toward the West is more recent. It was introduced into Latin America in the fifteenth century with the Arabian horses belonging to the Spanish conquistadores [35]. It was described for the first time on the Island of Marajo (Amazon estuary) in 1827, and was further observed in Paraguay (1847) in Pantanal, Brazil (1850), and Mato Grosso, Brazil (1860), before spreading into Bolivia, Venezuela, Guyana, and Colombia; it is present in Central America up to Mexico [4]. Nowadays, epizootics due to *T. evansi* are described periodically from Argentina to Panama [36], a geographical distribution related to the vampire bat *Desmodus rotundus*, a new host-vector reservoir of the parasite [4].

*Trypanosoma evansi* recently arrived in the Canary Islands (Spain) where it has been regularly observed since 1995 [37, 38]; it is thought to have been imported there by illegal introduction of camels from enzootic countries such as Mauritania or Morocco.

Toward the North, *T. evansi* was recently introduced on the Spanish mainland, in the Province of Alicante, where an outbreak occurred in a mixed camel and horse farm [39]. It was also introduced into France, in 2006, in a single epizootic focus in camels imported from the Canary Islands [40, 41]. These incursions into Europe should lead the sanitary authorities to include *T. evansi* among the animal health conditions for international trading of live animals within the European Union and other countries; thus, new procedures including diagnosis, curative or preventive treatment and quarantine should be established to ensure the status of these animals [42].

**Figure 1:**
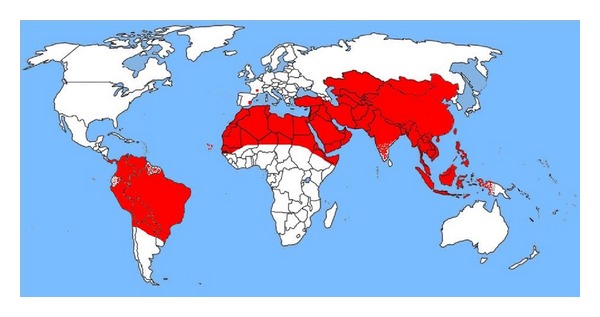
Geographical distribution of *Trypanosoma evansi *in the world (data synthesis).

The geographical distribution of surra is represented in Figure 1.

Historically, *T. evansi* could only be eradicated from areas where it was detected very early and controlled. Indeed, when introduced into America and Australia, in 1906 and 1907 [4], the infection was detected very early, during quarantine, and the animals were killed. In all the other cases, once *T. evansi* was established on an enzootic level, it was never eradicated, most probably due to the existence of a wide wild and domestic reservoir, the ability to be transmitted by nonspecific mechanical vectors present all over the world, and its ability to diffuse silently *via* healthy carriers. In such conditions, a reduction of the infected areas is not expected; on the contrary, the geographical spread of the parasite can be predicted.

In fact, the evolution of the geographical distribution of *T. evansi *is related to the movements of infected animals. Inside an infected country, the circulation of the parasite is almost free, especially with healthy carriers such as bovines, and also with more susceptible animals such as camels and mules, carrying the parasite with mild or subclinical signs. From one country to another, since the detection of the infection is sometimes impossible, infected animals may occasionally be allowed to enter uninfected areas, as was recently observed in the Canary Islands, and the Spanish and French mainlands [42].

Consequently, *T. evansi* is an unapparent spreading parasite.

## 3. Disease Synonyms and Parasite Taxonomy

*Trypanosoma evansi* belongs to the genus *Trypanosoma*, subgenus *Trypanozoon* (salivarian section) together with

- *T. brucei brucei*, one of the agents of a disease called Nagana in livestock, and for which wild animals often act as a reservoir; Nagana is a complex of diseases due to a number of *Trypanosoma species* including mainly *T. brucei brucei*, *T. vivax,* and *T. congolense* which have a great impact on cattle breeding in Africa;
- *T. brucei rhodesiense *and *T. brucei gambiense* are responsible for Human African Trypanosomiasis (HAT) or sleeping sickness, to which 60 million people are exposed in 36 sub-Saharan African countries; 70,000 persons are thought to be infected [2, 43] and the disease is most often fatal in the absence of treatment;
- *Trypanosoma equiperdum*, which is sexually transmitted in Equidae and is responsible for a disease called dourine.

The word “surra” comes from the *Indi* and means “rotten,” which qualifies the state of the animals after chronic evolution of the disease [28]; this especially fits to the evolution of the disease in camels. *Trypanosoma evansi* and surra are found under various names; Hoare reviewed the literature and found the parasite under more than 30 names [44], while the disease was found under an even greater number of vernacular names. In Venezuela: *T. equinum* or *T. venezuelense *was found to be the agents of *Peste-Boba* or *Derrengadera* (which means “limping”), in relation to nervous clinical signs in horses; in Argentina* T. hippicum* was found to be responsible for *Mal de Caderas*, in relation to the posterior paralysis of the legs, before the single name of *T. evansi* was adopted; however the disease is still found under its local names world over such as *Murrina* in Central America.

In Africa, for example, surra is found under the Arabic name* Debab *(*El debab* in Algeria) which means fly (linked to the vector) [45], and also *Mbori* in Sudan, *Guifar* or *Dioufar* in Chad,* Menchaca *(which means “emaciated” despite sufficient food provision) in Touareg populations of the Agadez area, Niger [46], *Yudleye* or *Yudle*, which refers to an emaciated camel aimlessly moving or jolting forward, or even* Dukhan * or *Salaf* (or *Salef*) in Somali [47] or Tahaga and su-auru [44]. The parasite itself was found under various names: *T. soudanense*,* T. marocanum*, *T. aegyptum,* and *T. cameli* before the single taxon *T. evansi* was accepted [4].

In Asia the name surra is mostly employed, although several other names were used before, such as *purana* (chronic or old), *tibarsa* (three-year disease), and *dubla* (emaciated) [32] or *makhi ki bimari* (horse-fly disease) [48]. However the name *T. evansi* was widespread, while in some areas it was found under other names such as *T. annamense* and *T. kirdanii* [4].

It is generally admitted that *T. evansi* derives from *T. brucei *through the complete loss of the maxicircles of kinetoplastic mitochondrial DNA, which are required to undergo the procyclic form in tsetse flies [27]. Losing in consequence its ability to perform oxidative phosphorylation [49], *T. evansi* is no longer able to undergo its cycle in Glossina [25–27], and it is “trapped” in its blood stream form. *T. evansi* also possesses only a single or very predominant minicircle sequence class [50]. A complete loss of the kinetoplastic DNA might even be possible and lead to akinetoplastic or dyskinetoplastic *T. evansi* which are observed in field stocks, but the use of some trypanocidal drugs may also enhance or even induce the rate of dyskinetoplastic forms [49, 51].

*T. equiperdum,* a parasite of horses, is closely related to *T. evansi. *It is sexually transmitted and responsible for a disease called “dourine.” It is also thought to be derived from *T. brucei* by an alteration of the kinetoplastic DNA, but maxicircles are still present in *T. equiperdum*, but with a single or very predominant minicircle sequence class [50]. Distinction [52] and even existence of this parasite are nowadays questioned since genetic differentiation is almost impossible, especially due to the absence of satisfying reference strains of *T. equiperdum* [53], but the distinction is still clear when looking at the minicircle complexity, which is very high in *T. brucei* (hundreds of minicircle sequence classes) and scarce, if any, in *T. evansi* and *T. equiperdum*. This diversity is most probably linked to sexual recombination, which can only occur in Glossina [54]. In recent decades in Europe, dourine has not been observed since 1994, though a recent outbreak occurred in Italy in 2011 [55, 56]; this may be an opportunity to study a recently isolated genetic material and provide some more conclusive data.

Although a number of authors have attempted to genetically characterize *T. evansi* and even to establish classifications [57–60], no convincing or useful classification has ever emerged. Even its distinction from *T. equiperdum* is sometimes questioned [52, 53]. The *Trypanozoon* subgenus constitutes a homogeneous group [61] and, especially inside *T. evansi*, most authors demonstrate a high molecular homogeneity [62], even though some reports state surprising heterogeneity at strain level [63, 64].

In several instances it was suggested that *T. evansi* and *T. equiperdum* should be renamed as *T. brucei evansi* and* T. brucei equiperdum* [36]; this suggestion was recently renewed based on the idea that these “subspecies” are *petite* mutants of *T. brucei* by deletion of genetic material [27]. However these technical considerations, which measure genetic divergence, neglect the most important concern we may have for these parasites: their pathogenicity, vectors, and host range and the consequent geographical distribution. In that sense, it seems reasonable and less confusing to keep the taxonomy as it is by considering the particular parasitic niche of *T. evansi* [65] in relation to its strong biological, ecological, and medical differences from *T. brucei*. Indeed, other authors support the hypothesis of a unique or at least a common genetic origin for all *T. evansi* since they were able to identify a synapomorphic gene in the parasite [61]. It is therefore advisable to keep the current nomenclature of *T. evansi*, as suggested by Touratier [66, 67], especially since the trinomial nomenclature is not in accordance with the rules of the international code for zoological nomenclature. Modification of this nomenclature would be a confusing mixture of history, phylogeny, and priority that a binomial nomenclature of life is supposed to know and summarize.

It is fair to point out that the discovery of nuclear fission never led to the terminology of the atom being abandoned, although it is indeed fissile!

Some authors have suggested that the spread of *T. evansi* and *T. equiperdum* was due to the lack of kinetoplastic DNA [68], but so far, while the relation between inability to develop in tsetse flies and akinetoplasty or dyskinetoplasty is clear, the relation with the ability to be transmitted by mechanical vectors, or to be sexually transmitted, has not been confirmed. Losing kinetoplast does not transform *T. brucei* into *T. evansi* (or *T. equiperdum*). Most probably, once *T. brucei* had lost all, or part, of its kinetoplastic DNA, parasites were selected either by mechanical vectors (selection of the most prolific parasites in the blood of a given host due to the very low quantity of blood transferred) or by direct contamination (selection of the most invading parasites in genital mucosae), in order to give birth to *T. evansi* and *T. equiperdum*, as the best performers by mechanical and direct sexual transmission, respectively. These speculations have not yet had any genetic support and must therefore be considered as pure hypothesis for further genetic characterization. However, selection of predominant slender forms of parasites by blood-sucking insects has been suggested for a long time [69]. Clearly, since it was mathematically demonstrated that the efficacy of mechanical transmission is directly proportional to parasitaemia [70], biting insects favour the spread of the most prolific strains of parasites in each host species. This consideration should be included in the attempts to understand the derivation from *T. brucei* to *T. evansi* in camels.

So far, it is advisable to keep the names of *T. evansi* and surra for the parasite and the disease, which most probably initially developed in camels.

**Figure 2:**
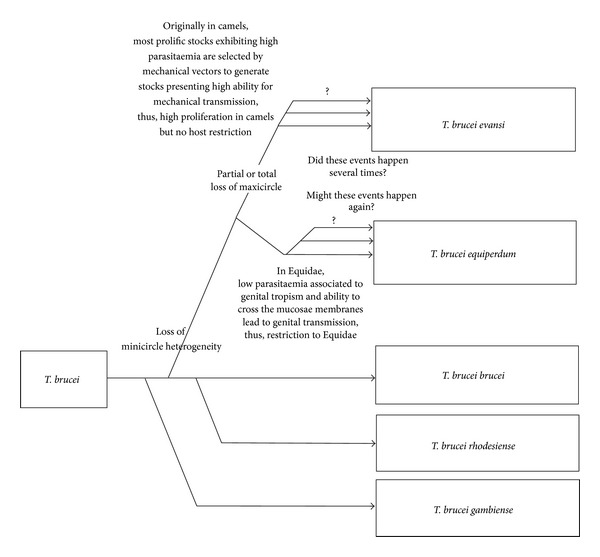
Hypothetical evolution tree for the *Trypanozoon* subgenus (data synthesis).

Be that as it may, a hypothetical evolution tree can summarize these data as presented in Figure 2.

Attempts to characterize *T. evansi* and *T. equiperdum* stock using random priming have led to some polymorphism being demonstrated among *T. evansi* strains, but so far it has not been possible to distinguish it from *T. equiperdum* [71, 72]. Other authors proposed RoTat 1.2 gene as a specific gene whose presence would characterize *T. evansi versus T. equiperdum*, but a number of studies have shown that this gene can be absent from some *T. evansi* stocks [58, 73–76]. So far, there is no single PCR test that can identify or distinguish between *T. evansi* and *T. equiperdum*. 

Lastly, although *T. evansi* could be considered as one of the 5 subspecies of *T. brucei*, under the name of *T. brucei evansi*, it seems justified to keep on using the species name of *T. evansi*, unless a trinomial nomenclature is accepted. A summary of the main characteristics of *T. evansi *is presented in Box 1.

## 4. Morphological Features of *T. evansi*

When observed in fresh blood samples, *T. evansi* presents the characteristics of slender *Trypanozoon* parasites: small size, compared with *Trypanosoma theileri*, but large compared to *T. congolense*, thin posterior extremity, free flagellum, active movements but producing limited displacements in the microscope field, and highly visible undulating membrane which “traps” the light (light may appear to be captured at one end of the parasite and transferred to the other end to be released).

**Figure 3:**
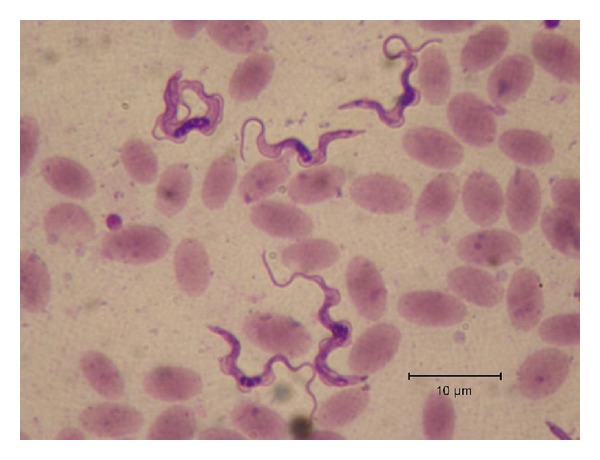
Morphological features of *Trypanosoma evansi*: classical forms in camel blood (M. Desquesnes). Legend: *T. evansi* in camel blood (France), Giemsa stained blood smear; typical morphology can be observed: large size (25–35 *μ*m), small and subterminal kinetoplast, thin posterior extremity, large undulating membrance, central nucleus, and free flagellum.

When observed on a Giemsa stained thin smear, *T. evansi* has always been described as a monomorphic thin trypomastigote parasite. By comparison with *T. brucei*, it shows mostly slender forms (long free flagellum and thin posterior extremity with subterminal small kinetoplast) (Figure 3) and some intermediate forms (shorter free flagellum and posterior extremity with almost terminal kinetoplast); however, there are some scarce reports of stumpy forms in this species, extensively studied by Hoare who concluded that the polymorphism of *T. evansi* is an inconsistent feature appearing sporadically [4].

**Figure 4:**
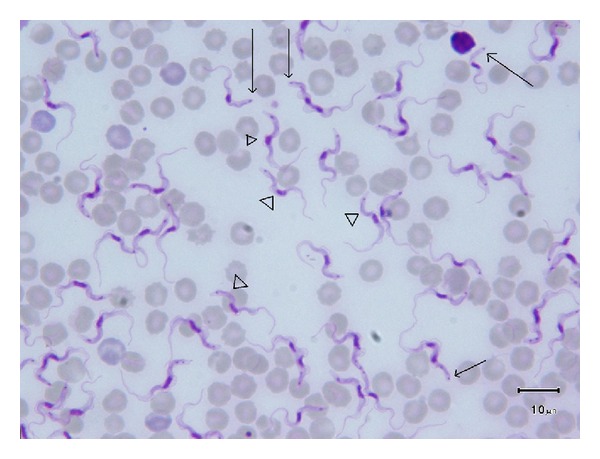
Morphological features of *Trypanosoma evansi *classical and truncated forms (M. Desquesnes). Legend: *T. evansi* in cattle blood (Thailand), Giemsa stained blood smear; typical morphology can be observed: with thin posterior extremity (head of arrows), together with truncated forms (arrows) whose posterior extremities are truncated just below the kinetoplast location.

The mean length of the parasite is 24 ± 4 *μ*m (min 15 *μ*m, max 33 *μ*m), without a sustainable relation between geographical, host, or even strain origin. Similarly, the morphological studies based on the absence of kinetoplast in a variable proportion of the population ranging from 0% (*T. equinum*) to 100% or intermediary (*T. venezuelense*) did not lead to any substantial distinction, and the dyskinetoplastic (or even akinetoplastic) strains are no longer regarded as different from *T. evansi*. Lastly, past and recent observations conclude that the size and shape of the blood forms of *T. evansi* are not in relation with genetic characteristics, but more or less with the growing conditions of the parasite and the immune response of the host [77]. It must be emphasized that in some instances truncated forms of the parasite are observed (Figure 4), and they may be confusing for species identification on blood smears, since the truncated parasite may look alike *T. vivax* as observed in the recent case in Spain [39]; however, the kinetoplast is larger in *T. vivax* than in *T. evansi*.

 To conclude, *T. evansi* exhibits the slender morphology and morphometry of the subgenus *Trypanozoon*, with very limited polymorphism and without any characteristics qualifying at species level.

## 5. The Large Host Range of *T. evansi*

*Trypanosoma evansi* has the widest host range amongst salivarian trypanosomes. It is especially pathogenic in camelids and equids. *T. evansi* also has a huge range of domestic and wild hosts worldwide. It has been hypothesized [26] that the loss of maxicircle kinetoplast DNA was responsible for the large range of hosts of *T. evansi, *but the same effects did not lead to the same results in *T. equiperdum* since, for the latter, the loss of maxicircle kinetoplast DNA [78] has led to a much narrower range of hosts. So it is still not understood why *T. evansi* benefits from so large a range of hosts, unless it is only a consequence of its geographical spread, which suggests that *T. brucei brucei* would have the same large range of hosts if it were able to spread outside the tsetse area. At least experimentally, almost all mammals are receptive to *T. evansi*, but only some of them are susceptible and may develop significant clinical signs and play a role in its epidemiology, as described below.

While almost all mammalian species are receptive, their susceptibility not only is highly variable from one species to another but may also be variable from one geographical area to another. For this reason we based the description of its host range on geographical units.

### 5.1. In Africa and the Middle East

*Trypanosoma evansi* is mainly a parasite of camels (*Camelus dromedarius*), the host species in which it probably early developed from *T. brucei brucei*. However, it is pathogenic in other Camelidae, such as the Bactrian camel (*Camelus bactrianus*). *Trypanosoma evansi *is highly pathogenic in Equidae, especially in horses (*Equus caballus*), and also in asses and donkeys (*Equus asinus*) together with their crossbreeds (mules), in which it is responsible for a sometimes acute disease, but most often chronic. 

*Trypanosoma evansi* can infect cattle (*Bos taurus*) in Africa; however they are sometimes refractory to the infection [79]. *Trypanosoma evansi *can affect pigs (*Sus scrofa*), domestic sheep (*Ovis aries*), and goats (*Capra hircus*). It is considered as nonpathogenic in the African buffalo (*Syncerus caffer*), in the serum of which a trypanolytic component was recently demonstrated [80]. *Trypanosoma evansi* is occasionally found in domestic cats (*Felis domesticus*) [81], and regularly in dogs (*Canis familiaris*), which may act as sentinel animals as observed in the surroundings of slaughter houses, since they can acquire the infection when eating fresh raw meat from infected animal. To conclude, in Africa, *T. evansi* is mainly a parasite of camels, which act both as the main host and a reservoir; it is sometimes found in horses and dogs, in which the infection is most often fatal.

### 5.2. In Asia

*T. evansi* is a major parasite for water buffaloes (*Bubalus bubalis*); in the Philippines it is considered as an economically important disease which concerns not only horses and buffaloes but also cattle, pigs, and goats [82]. In Asia, cattle are more receptive than in Africa or Latin America, and they can exhibit strong clinical signs [83] and very high parasitaemia (>10^8^ parasites/mL) can occasionally be observed in peripheral blood (unpublished observation). 

*Trypanosoma evansi* has been found in elephants (*Elephas maximus indicus*) in India where it affects them for work [44]; it has also been found in sick elephants in Thailand [84] where some seropositive animals were detected [83]. *Trypanosoma evansi* has been found in the antelope (*Saiga tatarica*), the sambar deer (*Cervus unicolor*), Rusa deer (*C. timorensis*) [85], hog deer (*Axis porcinus*) [86], barking deer (*Muntiacus muntjak*), chital deer, or spotted deer (*Axis axis*) [87] and in *Capreolus* spp. [44], as well as in wild sheep (*Ovis ammon*), wild pigs, tapirs (*Tapirus indicus*), rabbits, and pikas (*Ochotona pallasi*) and rodents such as *Rattus* sp., *R. tanezumi*, *Leopoldamys* sp.,* Niviventer fulvescens*, *Maxomys surifer *and *Bandicota* sp. [88, 89] and hamsters (*Cricetus cricetus*) [44]. Pikas and hamsters were found to be spontaneously infected in enzootic areas in Central Asia [4]. Mungos (*Herpestes javanicus*), the Indian hare or “Black nap hare” (*Lepus nigricollis*), the orangutan (*Pongo pygmaeus*), wolves, foxes (*Vulpes* sp.), jackals (*Canis aureus*), woodcats (*Felis bengalensis javanensis*), civet cats (*Paradoxurus*), badgers (*Helictis pierri* and *H. personatus*), and hyenas can be naturally or experimentally infected, and even chicks under experimental conditions [48, 90]. *T. evansi* has been found in leopards (*Panthera pardus*), jaguars, (*Panthera onca*), and tigers (*Panthera tigris*) in India [91–93]. *T. evansi *was recently observed in Asian rhinoceros (*Dicerorhinus sumatrensis sumatrensis*) in Malaysia [94] and in the Himalayan black bear (*Selenarctos thibetanus) *[95]. It has even been reported in chickens, but this single observation needs to be confirmed [96]; however the experimental infection of chicks has been demonstrated for a long time [48] and that of young pigeons more recently [97].

### 5.3. Surra in Australia and Europe

As it is able to infect deer, wild pigs, and rodents [88], *T. evansi* can become established in wild reservoirs all over the world, at the opportunity of infected animal movements. *Trypanosoma evansi* was introduced through infected horses in Australia and Canada in the early XXth century, but control measures, including slaughtering of infected animals, enabled early eradication [4]. However, *T. evansi* is a huge threat for Australia since it can affect horses, cattle, and camels (the latter, mostly returned to the wild, would be especially difficult to control). In addition to these traditional hosts, a possible role of several wild animals from Australia was studied in order to evaluate the risk of *T. evansi* dissemination from Papua New Guinea; wild pigs and Rusa deer proved to be receptive but of low susceptibility [34], while wallabies (*Macropus agilis* and *Thylogale brunii*) proved to be highly susceptible and exhibited acute clinical signs of surra, in most cases leading to death within 8–61 days [98]. Similarly, the Japanese vole (*Microtus montebelli*) proved to be highly susceptible since all 16 animals experimentally infected died [99]. In all cases, the potential for *T. evansi* to invade and establish as enzootic disease in Australia, Japan, or even Europe is a true and real threat. *Trypanosoma evansi* was introduced into the Canary Islands, most probably from Mauritania or Mali, in camels, and has yet to be eradicated [37, 100, 101]; from there it was introduced into continental Spain and France [40, 41]. In France it was controlled early and eradicated, but in Spain the situation remains unclear since camels, and also horses, were involved in the Alicante province [39, 102]. 

### 5.4. In the New World

*T. evansi* is found in host species introduced by humans, such as horses, cattle, buffaloes, sheep, and goats, but it has also been found in a very large range of local wild hosts. It is principally pathogenic in horses, sometimes with a very high prevalence, reaching 73% to 83% in the outbreaks reported from Brazil or Guyana [9, 103, 104]. It is found in water buffaloes with a prevalence reaching 40% in some instances [103, 105]; however, in the past, clinical signs of trypanosomosis in buffalos have most often been reported in infections due to *T. vivax* [106–110], rather than *T. evansi*. It has been reported in cattle with a prevalence of around 10% in Brazil, but there are no reports of pathogenic effects. *T. evansi* is regularly found in dogs, which are also infected by *T. cruzi*, and sometimes leishmania [111]; several reports from Guyana mentioned ocular haemorrhages and death with cardiac signs [9]. Guinea pigs (*Cavia porcellus*) can harbour the parasite, specifically in Peru, where they are raised for meat.

In addition to domestic hosts, *T. evansi *has been found in a large range of wild hosts.

The Latin America vampire bat (*Desmodus rotundus*) is simultaneously a host, reservoir, and vector of *T. evansi*; its role in the epidemiology of *T. evansi* is therefore crucial since it can not only transmit the disease but can act as a true reservoir, keeping the parasite in the bat colony in the absence of the main host [35].

Capybara (*Hydrochoerus hydrochaeris*), the biggest rodent in the world, wild or raised under free-ranging or semifree-ranging conditions, is potentially a major reservoir [112, 113]; in a study in Brazil, capybaras proved to be of low susceptibility and did not develop any anaemia [103]; in a study in Venezuela, 25% to 70% of the animals were found to be antibody carriers [114, 115]; a mathematical model to study the dynamics of transmission and spread by capybaras was recently developed [116].

Amongst camelids, *Lama glama* and *Lama pacos* are sometimes found to be infected; under experimental conditions *Lama guanicoe *proved to be fully receptive and susceptible to infection [117].

Infections have been detected in South American coatis (*Nasua nasua*), sometimes with a prevalence as high as 16% [103], wild dogs (*Canis azarae*), red howler monkeys (*Alouatta seniculus *and *A. ursina*), white tail deer (*Odocoileus virginianus chiriquensis*), brocket deer (*Mazama satorii*), wild pigs (collared peccary, *Tayassu tajacu,* and white-lipped peccary, *Tayassu pecari*), New World mouse (*Oryzomys *sp.), ocelots (*Leopardus pardalis*) [9], and armadillos (*Dasypus sp.*) as recently shown by PCR [118]. Marsupials such as the omnivorous *Didelphis* sp., *Monodelphis sp.,* and bats eating fruits and arthropods such as *Platyrrhinus* sp., *Carollia* sp., and *Myotis* sp. have also been found to be infected [103]; however their epidemiological role is not known.

Lastly, in Latin America *T. evansi* has been found in marsupials, Chiroptera, primates, lagomorphs, Edentates, rodents, carnivores, perissodactyls, and artiodactyls; however, the epidemiological importance of each species has not been determined and some may be epidemiological dead ends for mechanical vectors, due to very low parasitaemia rates [9, 36]. Nevertheless, these animals may still be a source of infection for carnivores. 

Finally, almost all mammals seem to be at least receptive, if not susceptible to *T. evansi*, and even some birds may be receptive; an exhaustive list of all potential hosts of *T. evansi* can therefore hardly be established. To complete the picture, a first, fully documented *human* case was recently reported from India [119], in a farmer who had fluctuating trypanosome parasitaemia associated with febrile episodes for several months; in the absence of central nervous system invasion, it was possible to treat the patient successfully with suramin. Contamination by contact of a wound with infected animal blood was suspected [120]. The potential of *T. evansi* to infect humans will be reviewed and discussed elsewhere.

## 6. Clinical Signs

The pathogenic effects of *T. evansi* are classical such as any other pathogenic mammal trypanosomes, including fever, anaemia, loss of appetite and weight, loss of condition and productivity, nervous signs and/or abortion, cachexia, and death, with or without more peculiar signs related to the host species [121]. However, what is quite surprising is the variable intensity of these signs, from totally unapparent to lethal, from one to another host species, but sometimes within a host species, depending on the geographical area or the epidemiological situation. Amongst nonvisible but very important effects of surra is immunosuppression, which will be presented in the next section.

Surra is basically a disease of camelids and equines, in which typical clinical expression is described, but various pathogenic effects are observed depending on the various domestic and wild hosts concerned. These signs by host categories will be described in this section, while the variations by geographical area and epidemiological situations will be detailed in the epidemiology section.

### 6.1. Camels and Horses

The typical clinical expression of surra can be described in camels and horses while donkeys, asses, and mules are of lower susceptibility.

Surra in camels (*Camelus dromedarius* and *C. bactrianus*) may be acute with high fever, anaemia, weakness, and death; it is also frequently fatal sometimes within a few months; however it is more often chronic than in horses and can frequently last 2-3 years (also called Tibersa) [122]. Signs of illness appear with intermittent fever (41°C), approximately about a week; the animals appear dull and lustreless and become progressively weaker with staring hair, loss of appetite and weight, abortion, oedema (ventral parts, udder or scrotum, and sheath), anaemia with pale mucous membrane, and petechial or ecchymotic haemorrhages. All the age groups can be infected but surra generally starts occurring shortly after weaning. Nervous signs are sometimes observed, such as periodic convulsions. The disease can last for several years and it is thought that they will recover if they survive more than 3 years. A specific odour of the urine is detected by camel owners, which is efficient for diagnosing the disease [44].

**Figure 5:**
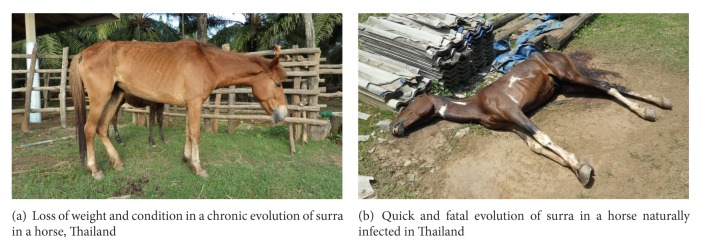
Chronic (up) and acute (down) evolution of surra in horses (M. Desquesnes).

**Figure 6:**
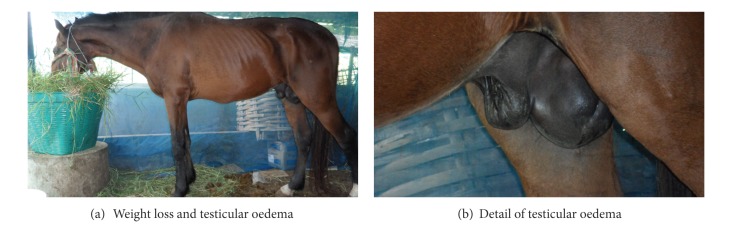
Weight loss and testicular oedema in a horse infected with *T. evansi *in Thailand (M. Desquesnes).

In horses, the incubation period is 1–4 weeks, and sometimes up to 8 weeks, after which the following symptoms appear: fluctuating fever with high peaks with parasitaemia (41.5°C up to 44°C), weakness, lethargy, anaemia, severe weight loss (Figure 5(a)), transient local or general cutaneous eruption, petechial haemorrhages on the eyelids, especially the nictitating membrane (which may turn yellow when reaching the icteric stage), vulvar and vaginal mucosa, haemorrhages into the anterior chamber of the eye (where trypanosomes can be also found in gelatinous material from the inner canthus), abortion, and alteration of locomotion, with nervous signs classically described in horses such as “it may stumble at the fore legs and drag the hind legs” [44], which probably called “*Mal de Caderas*”, and oedema (submaxillary, legs, briskets, abdomen, testicle and sheath or udder) appears after some time (Figure 6).

 In chronic evolution staring hair and a progressive loss of weight, which can lead to “living skeletons” as described by Evans, despite quite a conserved appetite, can be seen, but other authors mention a loss of appetite [104]; emaciation is often accompanied by jaundice and highly coloured urine [44]. Unless treated with trypanocidal drugs (diminazene aceturate, isometamidium chloride, quinapyramine, suramin, or cymelarsan), the disease can lead to death within 2–8 weeks. Animals can either die suddenly and unexpectedly or exhibit signs of delirium and struggle for hours before they die of exhaustion (Figure 5(b)). *T. evansi* is present in both intra- and extra-vascular fluids [123] which, together with regular changes of its variable surface glycoprotein (VSG), produce frequent relapses of parasitaemia and remittent clinical signs. Intravascular coagulation is thought to be responsible for persistent erection of the penis (Lingard 1893 quoted by [44]). 

There are considerable differences in the severity of syndromes caused by *T. evansi* depending on the virulence of the strain and the susceptibility of the host, but acute signs are often seen in naive populations with high mortality rates above 50% [104]. On the other hand, in enzootic areas, horses may exhibit a certain resistance with chronic or subclinical cases and healthy carriers. Donkeys and mules exhibit the same symptoms but milder than those in horses.

### 6.2. Cattle and Buffalo

Trypanosomosis due to *T. evansi* has long been considered as a mild, chronic, or asymptomatic disease in Bovinae (*Bos*, *Bubalus*, *Syncerus,* and *Poephagus*), especially in Africa and Latin America, where it is sometimes even difficult to infect animals experimentally [124]; similarly, in Venezuela, although some clinical signs have been recorded, the economic impact is not demonstrated [106]. 

The situation is quite different in India where the pathogenic effects of surra were recorded as early as 1891, with sometimes very high mortality rates (>90%), in reports fully documented by Gill [48] from numerous areas of India. Similarly, when surra was introduced in Mauritius the mortality rate was very high.

Experimental and natural infection of cattle with an Indonesian strain induced hyperthermia, haematocrit drops, and loss of weight [125–127] and could also lead to death [87], sometimes with nervous signs [128].

**Figure 7:**
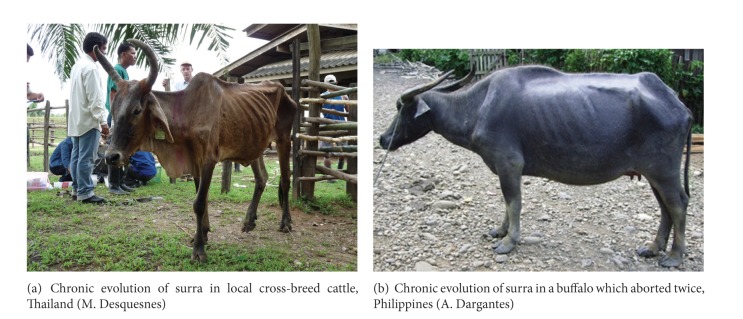
Chronic evolution of surra in cattle and buffalo.

In Asia in the last 3 decades, numerous reports have shown that surra is still, and maybe “again,” an important disease in cattle and buffaloes, especially in Indonesia, the Philippines, Thailand, and Vietnam [33]. Surra infection results in anaemia, losses in weight, milk and meat production, and losses in draught power, most often during chronic evolution which can lead to totally wasted animals (Figure 7(a)); occasionally the evolution may be acute, quickly leading to death. Indeed, fever, anaemia, abortion, and reduction in body weight gain leading to the interruption in oestrous cyclicity have been recorded in heifers in Indonesia [125]. In Thailand, the clinical signs recorded in buffaloes are fever, stiffness, conjunctivitis, emaciation, oedema (swelling of legs), inappetence, dyspnea, anaemia, recumbency, diarrhoea, abortion, and death [85]. Nervous signs are sometimes recorded with meningoencephalitis [123]. Buffaloes imported from Australia were particularly susceptible to the infection [129]. Similarly in North Vietnam, in the 1978–1981 period, hundreds of outbreaks led to 10% death in buffaloes following massive imports of buffaloes from Thailand and Cambodia; serological surveys showed 10–40% positive animals; similar studies in Thailand led to 15–54% seropositives in cattle and buffaloes [130].

In buffaloes, two syndromes have been described in the Philippines: a wasting sickness lasting weeks or months and terminating in recumbency and death and an acute disease leading to death within hours [33, 131]; *T. evansi* was thought to be responsible for the death of 10% of the buffaloes within a few months. A very high rate of abortion (47%) was also attributed to trypanosomosis (Figure 7(b)).

In dairy cattle, fever, abortion, and decreased milk production are frequently reported [132, 133]; in beef cattle, when surra occurs for the first time in a new area, high mortality can be recorded [134].

In all cases, if the clinical signs recede, it is suspected that surra exacerbates other latent infections [48], which will be studied in the next section.

### 6.3. Sheep and Goats

Natural infection is generally considered as mild or asymptomatic in sheep [135]. In some cases, experimental infections can even fail, but in others they can lead to clinical signs, mainly fever (40°C), lack of appetite, and anaemia; during hyperthermia, modification of behaviour such as exhaustion or sudden aggressiveness has been observed; anaemia can recede after 2 months; parasitaemia is generally low (10^5^ parasites/mL) and decreases until undetectable for several months; however, under certain circumstances such as food restriction or transport stress, parasites can relapse into the blood and clinical signs reappear [136]. In experimental infection of Yankasa sheep with a Nigerian isolate of *T. evansi*, acute and chronic evolutions were observed, with fever, pale mucous membrane, epiphora, loss of appetite, emaciation, dullness, and rough haired coat; in acute evolution the animals died within 2 weeks; postmortem observation indicated enlargement of the spleen and lymph nodes [137].

Goats are also most often of low susceptibility [69, 138]; thus in experimental infections with a camel isolate from the Canary Islands they showed mild symptoms with a few episodes of fever in early infection and arthritis in the next 6 months; although low, parasitaemia remained persistent [139]. In the Philippines, experimental infection led to the observation of fluctuating fever, progressive emaciation, anaemia, coughing, testicular enlargement, and diarrhoea but not in all animals [140]. However, other reports mention moderate [141] but sometimes severe or fatal infections with fever, lachrymation, salivation, loss of appetite, and nervous symptoms (shivering and convulsion) followed by hypothermia and death [142]. Ocular lesions have also been recorded [143]. Finally, the susceptibility of goats seems to be occasionally high in some reports, but, under natural conditions, most of the reports mention mild clinical signs due to *T. evansi* in goats [44, 144].

As sheep and goats are not regular hosts of *T. evansi*, based on the reports available, it is difficult to decide on their susceptibility.

### 6.4. Pigs

Infection in pigs has long been reported as very mild or symptomless; however, symptoms such as fever, anorexia, emaciation, and abortion were reported in an outbreak in pigs in Malaysia [145], and there were reports of low fertility in Thailand [146]. Even under experimental conditions, clinical expression is mild or delayed for several months. The immunosuppressive effects of the parasite have been considered to be responsible for interference with the efficacy of the vaccine against Classical Swine Fever [24].

**Figure 8:**
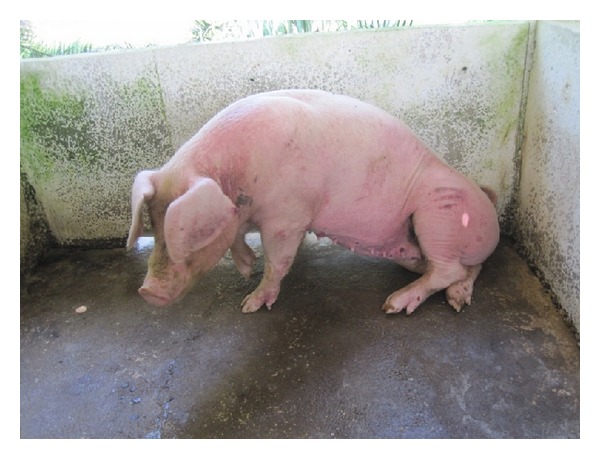
Hind leg paralysis in a pig naturally infected by *Trypanosoma evansi *in Malaysia (courtesy, Dr. Chandrawathani Panchadcharam).

Pig infection is often chronic with not only intermittent fever, anaemia, loss of weight, abortion, and cutaneous rash, but also late nervous evolution, with hind leg paralysis (Figure 8). While most of the reports are mild cases, there are a number of reports of severe outbreaks in Thailand; in Chachoengsao province 85% of the animals were infected [147] and relapsed after treatments with isometamidium chloride; in Phitsanulok province, in 1984, on a sow and boar farm, a severe outbreak was reported, with fever, anaemia, urticarial plaques on ventral parts of the body, around teats and udders or scrotum, lateral parts of the body and ears, and even nervous symptoms of convulsion and circling [148]; in Nakhon-Pathom province, in 1982, 19/22 sows showed clinical signs of surra with fever (39–41°C) and abortion [149]. In an experimental report cutaneous signs and abortion were observed in sows [150]. It seems that, similarly to goats and sheep, some rare outbreaks of surra may be severe in pigs, but the reasons for these outbreaks are not known. Finally, though little attention has been paid to surra in pigs, these reports suggest that surra may have been underdiagnosed and then underestimated in this species.

### 6.5. Carnivores

**Figure 9:**
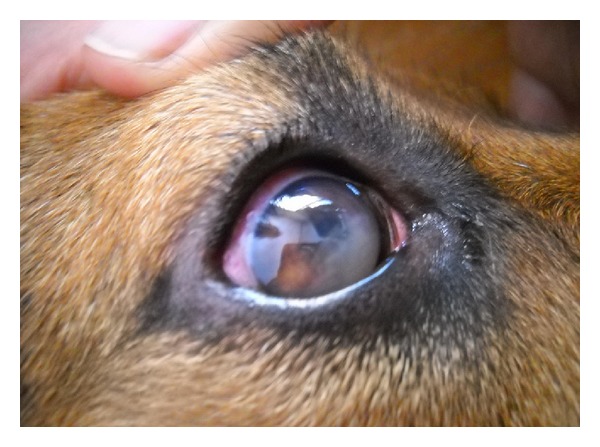
Fibrin deposit in the anterior chamber of the eye, in a mixed German shepherd, naturally infected by *Trypanosma evansi*, Chiang Mai, Thailand (courtesy Miss April Terry).

Dogs are highly susceptible to *T. evansi*, and they often exhibit strong clinical signs leading to death, sometimes within a week and most often within a month in acute cases [48], especially in stray dogs which are not treated [103] and also sometimes even despite treatments [151]. Clinical signs are intermittent fever (39°C–41°C), oedema of the head, including larynx (to be differentiated from rabies), oedema of the abdominal wall and legs, anaemia, weakness, lack of appetite leading to emaciation and, sometime, paresis of the hindquarters; myocarditis has been described and can be fatal, as described in the first record of *T. evansi* in French Guiana [9]; sexual excitement has also been mentioned. Ocular signs are most often observed in dogs, with conjunctivitis, lachrymation, keratitis, corneal opacity, and/or haemorrhagic signs, which can lead to fibrin deposits in the anterior chamber of the eye (Figure 9); parasites have sometimes been observed in ocular aqueous fluid; these signs can recede after treatment in some instances [111, 136, 152–154]. Most of the cases are related to hunting dogs or dogs living around slaughter houses, which suggests peroral infection; however, seasonal effects have also been recorded [151]. Transmission by stomoxes, which is the other name for dog fly, is also possible providing the dog is living in close contact with another infected animal.

Very little is known about natural infection in cats, but *T. evansi* experimental infection in cats induced only mild symptoms, such as fever, apathy, hyporexia, and vomiting [155] as well as muscular pain, hyperproteinaemia, hyperglobulinaemia, and hypoalbuminaemia [156].

Other carnivores have been found to be infected and susceptible, such as ocelots (*Felis pardalis*), tigers [157], hyenas, and leopards [158].

### 6.6. Other Naturally Infected Domesticated Species

In the Asian elephant, severe symptoms are observed with fever, anaemia, anorexia, oedema of the face, trunk, neck, brisket, lower abdomen and limbs, dry and hard skin, sluggish movement, dullness, restlessness, sleepy moods, reluctance to work, ecchymoses, conjunctiva, and a high mortality rate in Myanmar (Burma) and India [44]. In Thailand, fatal [159] or moderate cases have both been described [84]; treatment with diminazen aceturate gives irregular results with some failures using 5 mg/Kg [159] and some successes using 8 mg/Kg [160], but in the latter case, elimination of the parasite could not de demonstrated. 

In deer, several reports gathered by Gill (1977) showed variable signs depending on the host species; acute and fatal evolutions were observed in *Antilope cervicapra* and Axis sp. while it was more chronic in *Axis axis* and *Rusa timorensis*, with anaemia, loss of weight, and abortion. Acute signs were reported from outbreaks into Mauritius, in *Cervus unicolor*, with acute fever, rapid loss of condition, emaciation, anaemia, and death [48]. In South China a 20% death rate was recorded on a deer farm [161]. In Thailand, in *Cervus porcinus* (hog deer), nervous signs were reported with paresis, lateral recumbency, excitation, convulsion, and a high mortality rate; presence of *T. evansi* in the Virchow-Robin spaces of the brain was demonstrated by immunohistochemistry [86, 162]. Similarly, an outbreak in the Java deer (*Cervus timorensis*) was reported from Malaysia (Perak) with anaemia, inappetence, respiratory distress, recumbency, and lethal evolution; in this case several other haemoparasites were present together with *T. evansi* infection [163]; in total during this outbreak, surra was responsible for a 27% mortalityrate [164].

### 6.7. Wild Hosts

Surra is classically described in a number of favoured wild hosts such as vampire bats, capybaras, and coatis; in the latter, experimental infections revealed the existence of serious anaemia, myocarditis, and meningoencephalitis [165]. *Trypanosoma evansi* is also present in a large range of other wild animals including wild pigs, deer, and rodents, which are mostly healthy carriers. However, more susceptible host species have been identified recently.

Experimental infections have been carried out and have demonstrated that a number of other species are receptive and susceptible to the parasite. Amongst them, the wallaby, which is the most common species of macropodid in southern Papua New Guinea (PNG) and northern Australia, was experimentally infected to test the potential for the spread of surra in PNG and Australia where other potential hosts are abundant, such as feral pigs and Rusa deer [34]. Agile wallabies (*Macropus agilis*) and dusky pademelons (*Thylogale brunii*) both proved to be very susceptible to the infection; they developed high parasitaemia 6 days after infection, persisting until death, between 1 week and 2 months; clinical signs were anorexia, weakness, ataxia, and anaemia, while the autopsies revealed pericarditis, splenomegaly and ulcerative gastritis and enteritis [98].

*Trypanosoma evansi* was observed in 4 natural infections in Himalayan charming bears, in Pakistan; the animals exhibited pyrexia, accelerated pulse, tachypnea, depression, anaemic mucous membranes, and ataxia [95].

Surra was suspected in 5 captive Sumatran rhinoceroses (*Dicerorhinus sumatrensis sumatrensis*) in Malaysia presenting depression, anorexia, incoordination, muscle tremor, nasal haemorrhage, recumbency, and labored breathing followed by death. *Trypanosoma evansi* was found in 3 out of 5 animals, which all died [94]. 

*Trypanosoma evansi* was suspected in a herd of Arabian dorcas gazelles (*Gazella dorcas saudiya*) and in one Sand gazelle (*Gazella subgutturosa marica*) in Kuwait; the main clinical signs were paresis of hindquarters and sudden death; successful treatment was obtained with melarsomine (http://priory.com/vet/Trypanosomagazelles.htm).

## 7. Immunosuppressive Effects

Trypanosomes survive and multiply in the extracellular fluids of their mammalian hosts, especially in the blood. They are thus confronted with both innate and adaptive immune defences. Selective pressure has thus enabled them to elaborate refined escape mechanisms. Besides its direct pathogenicity, sometimes limited, but visible from clinical or paraclinical observation, the impact of trypanosomiasis lies in the ability of parasites to cause immunosuppression, which is a dual biological phenomenon: on the one hand it prevents immunopathology that can injure the host (synergism among proinflammatory cytokines was demonstrated to contribute to the development of anaemia [166]), but on the other hand, it allows a small trypanosome population to evade the protective immune responses, remaining clinically silent in the host further playing the role of a zoonotic or anthroponotic reservoir. Immunosuppression also reduces the efficiency of host immune responses, leading either to the development of intercurrent diseases or depreciating the quality of vaccine immunity. This immunopathological aspect was highlighted in the early seventies [167] but seems to be speciesdependent as demonstrated in murine experimental models, and *T. evansi* seems to have developed particular strategies when causing surra. The most well-known escape mechanism developed by trypanosomes is the antigenic variation by which they successively exhibit various main membrane surface glycoproteins: the variant surface glycoprotein (VSG). This can be considered as a first intention immunosuppression; however it proceeds from immunological exhaustion, since trypanosomes force their host to elicit successive directories of antibodies able to cope with emerging VSG variants, while a new variant is planned to develop before the humoral response is effective [168, 169]. Interestingly, a skin test in rabbits infected by *T. evansi* demonstrated an immediate type hypersensitivity reaction, followed by a delayed type against the parasite surface-associated components, which exhibited more intensity in cured animals than in infected ones. This supports both VSG-specific antibody activity and cellular immunosuppression [170]. Immunosuppression can paradoxically be a consequence of an exacerbated inflammatory reaction initially developed to control parasitaemia, as demonstrated by high levels of acute phase proteins (C-reactive protein, haptoglobin, and alpha 2-macroglobulin) concomitantly with immunoglobulins (Ig) M targeting VSG [171]. Inhibition of blood acetylcholinesterase activity, an inflammatory marker in acute and chronic *T. evansi* infection in rabbits, resulted in improved immunological response against trypanosomes by proinflammatory cytokines [172, 173]. A consequence of inflammatory response is the increase in extracellular adenine nucleotides such as ATP, which are normally hydrolysed into AMP by ectoenzymes such as NTPDase (EC 3.6.1.5, CD39). One of the immunosuppression characteristics induced by *T. evansi* and linked to inflammation was the altered NTPDase activity on the surface of lymphocytes of infected rats [174].

The complement system is one of the first molecular defences in innate immunity, and antibody-dependent complement-mediated lysis is probably one of the most efficient early control strategies developed by the host. Unfortunately, data from experimental infections in camels indicated that, despite a slight initial increase, classical complement pathway haemolytic activity dropped as the infection progressed and correlated negatively with parasitaemia but was recovered following elimination of trypanosomes, strongly suggesting an immunosuppression of the molecular components of the immune system [175]. In terms of innate cell-mediated immune response against trypanosomes, macrophages play a central role as antigen presenting cells (APCs) and effector microbicidal cells. Trypanosomes modulate macrophages through parasite factors and host cytokines to control cell polarization into distinct activation states (M1, M2), which may further contribute to susceptibility or resistance to infection [176, 177]. Trypanosome killing is assumed to occur via the induction of classically activated macrophages (M1-type macrophages) that produce high levels of inflammatory compounds such as tumour necrosis factor *α* (TNF-*α*), reactive oxygen intermediates, nitric oxide synthase 2-dependent reactive nitrogen intermediates, such as NO and associated molecules [177]. Interestingly, in murine models of *T. evansi* trypanosomosis, whereas infection causes the induction of interferon *γ* (IFN-*γ*), TNF-*α*, and NO, none of these molecules was found to be crucial for parasitaemia control and survival of the infected animals [178]. A trypanosome-suppressive immunomodulating factor (TSIF) was shown to induce TNF and NO secretion by M1 macrophages, which concomitantly blocked T cell proliferation in a NO- and IFN-*γ*-dependent manner. Furthermore, TSIF had the capacity of downregulating type 2—oriented immune responses, being a key molecular actor of the trypanosome-induced immunosuppression [179]. This largely explains the elevated NO levels found in the serum of rats infected by *T. evansi*, associated with a redox imbalance (advanced oxidation protein products (AOPP) in serum and superoxide dismutase (SOD) and catalase (CAT) activities in blood) [180]. Moreover, this could be linked to one of the main characteristics of trypanosome-induced immunosuppression in both experimental rodents and natural hosts, which consist in the eliciting of suppressor macrophages that results in a NO-mediated unresponsiveness in lymphocytes. In that way, IFN-*γ*- and TNF-*α*-dependent NO production could be involved in the suppression of splenocyte proliferation occurring in *T. evansi* infection [178]. Amazingly, the apparent loss of suppressor macrophage activity in cured animals was shown to be due to NO-mediated apoptosis of these cells [181]. Among APCs, dendritic cells (DCs) are known to be strong elicitor and regulator cells of the immune system. Behind the inflammatory cytokine and chemokine storm caused mainly by macrophages in *T. evansi* infections, increased expression levels for Ccl8 and Il10 in splenocytes suggested an increase in the number and activity of regulatory dendritic cells (DCs). The regulatory DCs became prevalent during the progress of infection, therefore reducing the amount of inflammatory DCs, and as a potential regulator of the inflammatory responses, suggesting the use of the inflammatory responses to immunosuppress the host, but regulation to avoid irreversible pathophysiological effects [182].

Despite the elements described above, and contrary to tsetse fly-transmitted trypanosomes, the immunobiological disorders occurring during a *T. evansi* infection have been little documented, and the reports of immunological dysfunction occurring throughout the disease have only partially addressed the corresponding control mechanisms. In water buffaloes, *T. evansi* infection induced a significant decrease in haemoglobin concentration, packed cell volume (PCV) and red blood cell count, kidney function (creatinine and urea), and liver alkaline phosphatase, whereas total the leucocytic count, lymphocyte, and monocyte populations increased, as well as liver functions (lactate dehydrogenase enzyme (LDH) activity, globulin, total bilirubin, and indirect bilirubin), showing a direct link between immune and metabolic disorders [183–188]. In experimentally infected sheep, dissection of the immune components involved in *T. evansi*-induced immunosuppression highlighted that macrophages but not CD8(+) T cells were mainly responsible for suppression [189]. Actually, in terms of lymphocyte populations it was shown that an increase in the CD4 : CD8 ratio and IgG1 was associated with self-cure in *T. evansi*-infected sheep, whereas a decrease in the CD4 : CD8 ratio and IgM associated with an increase in the number of sIg+, CD45R+, CD1+, a major histocompatibility complex (MHC) II+ circulating B cells, was associated with infection and disease development [186, 190]. 

Recently, the relative contribution of IgG versus IgM antibodies was detected for *T. evansi* infection in mice; the absence of both B cells and IgM resulted in the abolishment of first peak parasitaemia control and consequently rapid death of the infected deficient mice [178]. Passive transfer of infection-induced IgG and IgM antibodies from normal mice to B-cell- or IgM-deficient mice confirmed that antibody-mediated *T. evansi* parasite control relied on IgM rather than on IgG, in contrast to what happens in *T. brucei* and *T. congolense* infections [178]. However, while existing in the case of *T. evansi*, it is not clear why IgM-mediated phagocytosis would be more efficient and protective than IgG-mediated phagocytosis of opsonised trypanosomes, which is classically reported [191]. Contrary to *T. brucei* and *T. congolense*, *T. evansi* exhibits distinct molecular and cellular dialogues and conflicts when interacting with a mammalian host, since despite an infection-associated induction of trypanocidal inflammatory molecules, only IgM antibodies were proved to significantly contribute to trypanosome control [177]. Moreover, to achieve immunosuppression of the host, even if demonstrated only with *T. brucei*, it has been proven that a nonrelated vaccine-induced protection was completely abolished during an ongoing trypanosome infection. Initially, this was attributed to active immunosuppression during infection. However, even after antitrypanosome treatment with Berenil, there was no recovery of vaccine efficacy against an infectious challenge. These results suggest that at least in a mouse model, trypanosomes are capable of permanently destroying the host B-cell memory compartment, in a nonantigen specific manner [192]. In the same way, it has been proved recently that a *T. evansi* lymphotoxin is able to induce CD45-dependent lymphocyte death [193], which correlates with pioneering findings demonstrating that membrane fractions of *T. evansi* elicit suppressor cells [194].

A disturbing consequence of the immunosuppression induced by the trypanosome is the highest level of chemoresistance achieved using cloned trypanosomes in immunosuppressed mice. By frequent passage in immunosuppressed mice given subcurative drug treatments, *T. evansi* was demonstrated to rapidly develop high levels of resistance to diminazene aceturate and isometamidium chloride, which did not happen in immunocompetent mice. Immunosuppression of animals by a heavy parasite burden or stressful conditions in conjunction with underdosing may therefore play an important role in the development of drug resistance under field conditions [195, 196]. Moreover, the quick degradation of the effectiveness of the host immune system induced by *T. evansi* may explain the deadlock in developing an efficient anti-trypanosome vaccine, despite the identification of several nonvariant surface-exposed trypanosome immunogens. More worrying is the loss of effectiveness of conventional vaccines used in farm animals demonstrated first in laboratory rodents, as illustrated for *Trichinella spiralis* [189, 190]. Moreover, surra is suspected to induce an immunosuppressive syndrome in cattle and buffaloes, indicated by a lost capacity to mount humoral and cell-mediated immune responses against heterologous antigens, which would be responsible for failures of the vaccination campaigns against foot and mouth disease (FMD) and haemorrhagic septicaemia (HS) [188, 197, 198]. It also affects sheep by delaying and depressing the number of lymphoblasts induced by *Pasteurella haemolytica* vaccine administration [199] as well as pigs by interfering with their immune response to Classical Swine Fever (CSF) vaccine [24]. Some experiments have provided an explanation of the cellular events linked to the loss of immunity, as significant increases in circulating CD5+ B cells associated with significant decreases in CD5+, CD4+, and CD8+ T cell subsets were observed in *T. evansi*-infected *Pasteurella haemolytica*-vaccinated sheep at the inoculation site. Cell population dysregulation was associated with suppression of local skin reaction and serum IgG1 antibody responses to the vaccine antigen [184]. The same results were obtained when performing the experiment and analysis on lymphocyte phenotypes draining from a lymph node of a *T. evansi*-infected *Pasteurella haemolytica*-vaccinated sheep, allowing the authors to conclude that these abnormal changes in the kinetics of efferent lymphocyte phenotypes are likely to play a role in the genesis of the generalized immunosuppression seen in trypanosome-infected hosts [185]. Lastly, the proliferative responses of *T. evansi*-infected *Pasteurella haemolytica*-vaccinated ovine peripheral blood leucocytes (PBL) Concanavalin A (Con A), bacterial lipopolysaccharide (LPS), *PASTEURELLA* antigen (P.ag), or homologous trypanosome antigen (T.ag) were significantly suppressed by the infection, but fully restored by trypanocidal treatment for Con A, LPS and T.ag only, whereas for P.ag the responsiveness of cells from uninfected vaccinated sheep remained significantly higher than those of cells from infected sheep [183]. This strongly suggests that the immunosuppression induced by *T. evansi* may have an even more dramatic impact, because the treatment of trypanosome infection would have no impact on the loss of protection against common animal diseases.

Nevertheless, in the mouse model, some studies tend to bring hope in the possibility of immunizing against *T. evansi* since some authors succeeded in protecting animals from trypanosome infections by immunisation against parasite proteins such as *β*-tubulin [200, 201], actin [202], and VSG [201–203]. Moreover, immunisation of mice with paraflagellar rod proteins (PFR) 1 and 2 evidenced trypanolytic properties of the anti-PFR1 and anti-PFR2 sera [204].

Immunosuppressive mechanisms occurring in *T. evansi* infections have been partially characterized in a number of models, from laboratory rodents to natural hosts, but they need to be further investigated and understood and would serve as a basis for studying infectious parasitic immunosuppression.

## 8. Conclusion

In this paper, we have overviewed the basic characteristics of *T. evansi*, including its origin, possibly multiple, which suggests that it might be a plural parasite (petite mutant) [27]. What happens exactly when *T. brucei* leaves Africa, and is submitted to selection which may be governed by venereal or mechanical transmission, is not fully understood yet [68] and would require a complete review of its genetics as well as those of *T. equiperdum*, a very close, if ever different, parasite [53]. As the molecular epidemiology of *T. evansi* is, in itself, a topic for a large review, it will be conducted elsewhere. However, and lastly, losing some genetic material (kinetoplastic maxicircles) made this parasite more efficient in terms of host and vector ranges. Indeed, when leaving the tsetse belt “jail” in which it was trapped by its cyclical development in tsetse flies, *T. evansi* developed, or simply expressed, a surprising and spectacular ability to develop in a very large range of hosts leading to a no less spectacular, potentially unlimited, geographical distribution. Although *T. evansi* has long been claimed to be a genetically and morphologically highly homogeneous parasite [205], recent investigations have demonstrated more diversity than expected [206]. Moreover, it is obvious, when comparing *T. evansi *to* T. brucei*, that some slight modifications in the nucleotidic sequence of a genome have deeply impacted the biological properties of a parasite, affording it very different characteristics and behaviours, both in terms of host range, pathogenicity, transmission, epidemiology, and geographical distribution. Initially developing in camels in North Africa, *T. evansi* had—via biting insects acting as mechanical vectors—iterative occasions to infect other mammal species living in the vicinity of camels. When occurring, these occasional infections might, or might not, lead to successful epidemiological systems, depending on the characteristics of vectors, hosts, environments, and animal management. This scenario suggests that successful attempts may have led to the development of “a number” of *T. evansi *due to selection through the hosts and the vectors. Successful associations of host and vectors of *T. evansi* resulted in a gradient of epidemiological systems, from camels, in North Africa, to cattle and equids in the Middle East, ending with water buffaloes in South-East Asia. Due to its potentially unlimited host range, *T. evansi* also possesses a capacity to invade new geographical areas, as shown by the recent incursions made in into continental Spain and France. Consequently, the scientific community and sanitary authorities should pay attention to this parasite, which may have opportunities for new developments in its geographical distribution, towards the North, both in Europe and America, or towards the South, in Australia.

Another important aspect of this parasite is the various clinical or subclinical evolutions that may occur in several hosts and/or areas. Camels, horses, and dogs remain the most critical hosts for this parasite. In camels, classically, the disease evolution can be acute, chronic, and subclinical, including healthy carriers; the chronic infection leads to name the disease as surra (rotten) *Menchaca* (emaciated) or *tibarsa* (three years disease). In horses, *T. evansi* induces an acute and most often fatal disease which temporary leads to giving new specific names such as *T. equinum* or *T. hippicum* before concluding on a single parasite exhibiting pleomorphic signs in a large range of hosts. *Trypanosoma evansi* can multiply with huge scores and then spread very quickly, and in a very efficient manner, through biting insects, towards other surrounding host species; the role of biting insects is pointed by local names such as *El debab* which (flies) or *makhi ki bimari* (horse-fly disease) in Algeria and Punjab, respectively. Moreover, in equids, weakness of the legs or even nervous infections have led to naming the disease *Mal de Caderas* or *Derrengadera* in South America. Although it can most often kill horses and thus destroy its own survival reservoir, these outbreaks are the opportunity to spread to other reservoirs. In such mixed epidemiological systems, the parasite can use equines to multiply and spread, while it can use bovines (cattle and buffaloes) as mild regular hosts and very efficient reservoirs. Other epidemiological systems have developed in parallel, such as mules/horses (tolerant reservoir host/acute outbreak substrate), or “peroral infected flesh to carnivore system,” or “infected flesh to wild rodents system” for which it is not yet understood whether they are epidemiological dead ends or potentially active reservoirs. The passage from carnivores or rodents back towards herbivores is not clear enough and would need more investigations to be clarified. Presumably the various degrees of pathogenic effect observed in the various hosts affected may reflect adaptations and/or selection of the parasites with regards to the hosts or the means of transmission (biting insects in herbivores or peroral infection in carnivores), which may explain the various features offered by *T. evansi* in different geographical or host specific situations.

The impact of the disease will be reviewed elsewhere, but given the data reported on clinical and immunosuppressive effects, it can be pointed out that the capacity of *T. evansi *to spread undetected, to induce immune or vaccination failure (thus impacting other disease developments or strategies such as FMD, HS, or CSF), or to be hidden behind other more obvious diseases such as anaplasmosis or babesiosis clearly suggests that it will also be necessary to pay more attention to this understated parasite.

## Figures and Tables

**Box 1:**
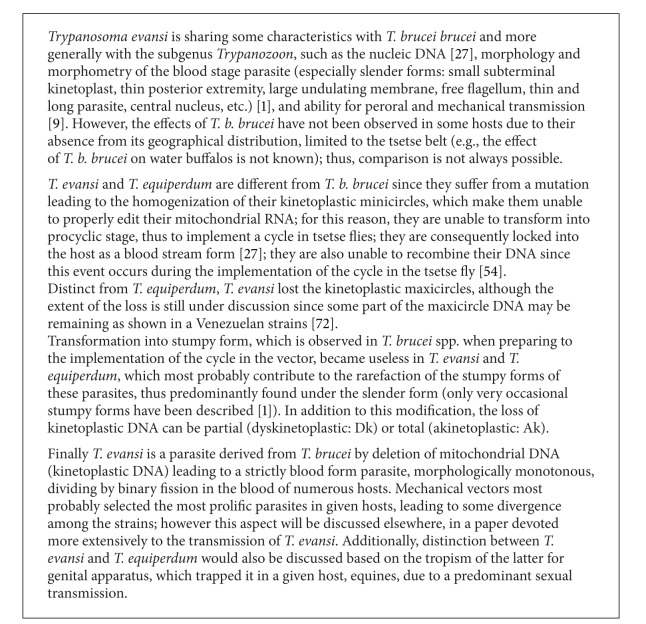
The main characteristics of *Trypanosoma evansi*.
